# Warmer temperatures interact with salinity to weaken physiological facilitation to stress in freshwater fishes

**DOI:** 10.1093/conphys/coaa107

**Published:** 2020-12-15

**Authors:** Richard H Walker, Geoffrey D Smith, Spencer B Hudson, Susannah S French, Annika W Walters

**Affiliations:** 1 Wyoming Cooperative Fish and Wildlife Research Unit, Department of Zoology and Physiology, University of Wyoming, 1000 E University Ave, Laramie, WY 82071, USA; 2 Biological Science Department, Dixie State University, 225 S 700 E, St. George, UT 84770, USA; 3 Department of Biology, Utah State University, 1435 Old Main Hill, Logan, UT 84322, USA; 4 US Geological Survey, Wyoming Cooperative Fish and Wildlife Research Unit, Department of Zoology and Physiology, University of Wyoming, 1000 E University Ave, Laramie, WY 82071, USA; 5 Program in Ecology, University of Wyoming, 1000 E University Ave, Laramie, WY 82071, USA

**Keywords:** Antagonistic interaction, cortisol, glucose, multiple stressors, sodium bicarbonate, stress reactivity

## Abstract

Management of stressors requires an understanding of how multiple stressors interact, how different species respond to those interactions and the underlying mechanisms driving observed patterns in species’ responses. Salinization and rising temperatures are two pertinent stressors predicted to intensify in freshwater ecosystems, posing concern for how susceptible organisms achieve and maintain homeostasis (i.e. allostasis). Here, glucocorticoid hormones (e.g. cortisol), responsible for mobilizing energy (e.g. glucose) to relevant physiological processes for the duration of stressors, are liable to vary in response to the duration and severity of salinization and temperature rises. With field and laboratory studies, we evaluated how both salinity and temperature influence basal and stress-reactive cortisol and glucose levels in age 1+ mottled sculpin (*Cottus bairdii*), mountain sucker (*Catostomus platyrhynchus*) and Colorado River cutthroat trout (*Oncorhynchus clarki pleuriticus*). We found that temperature generally had the greatest effect on cortisol and glucose concentrations and the effect of salinity was often temperature dependent. We also found that when individuals were chronically exposed to higher salinities, baseline concentrations of cortisol and glucose usually declined as salinity increased. Reductions in baseline concentrations facilitated stronger stress reactivity for cortisol and glucose when exposed to additional stressors, which weakened as temperatures increased. Controlled temperatures near the species’ thermal maxima became the overriding factor regulating fish physiology, resulting in inhibitory responses. With projected increases in freshwater salinization and temperatures, efforts to reduce the negative effects of increasing temperatures (i.e. increased refuge habitats and riparian cover) could moderate the inhibitory effects of temperature-dependent effects of salinization for freshwater fishes.

## Introduction

In an era of widespread anthropogenic change, conservation science requires a better understanding of how species respond to multiple, interacting stressors (Jackson *et al.*, 2016; Craig *et al.*, 2017). Salinity and temperature are two important physiological mediators for aquatic organisms with the potential to become chronic stressors if not properly managed (Uliano *et al.*, 2010). And the consequences of increased salinization and temperatures are likely contingent on their interactive effects (Wang *et al.*, 2019). Freshwater salinization from anthropogenic activities (e.g. road deicers, sewage, energy extraction, leaks and spills) and saltwater intrusion is becoming widespread—modifying ion composition and concentrations in many ecosystems (Gornitz, 1995; Kaushal *et al.*, 2005; Cañedo-Argüelles *et al.*, 2013). Similarly, increased temperatures associated with climate and land-use changes have already affected many freshwater ecosystems resulting in physiological challenges that have triggered shifts in species’ ranges, reproductive success and population abundances (Chen, 2011; Wenger *et al.*, 2011; Gibson-Reinermer *et al.*, 2015; Firkus *et al.*, 2018). While long-term biological responses, such as delayed growth and reduced survival of freshwater organisms have frequently been evaluated (Kinne and Kinne, 1962; Tay and Garside, 1974; Rogell *et al.*, 2009; Hopkins *et al.*, 2017), our understanding of how these stressors interact to affect short-term physiological responses remains limited (Powers, 1920; Hopkins *et al.*, 2017; Phuc *et al.*, 2017).

Expansion of oil and natural gas development (ONGD) poses numerous threats to freshwater organisms (Entrekin *et al.*, 2011), including increased water temperatures and salinization (Blair *et al.*, 2016; Davis *et al.*, 2009; Bern *et al.*, 2015; Walters *et al.*, 2019). For example, land-use change associated with ONGD (e.g. roads, pipelines, well pads, waste-water holding ponds and refining facilities) has been linked to increased sediments, sediment-bound contaminants, ions and temperatures in receiving streams (Williams *et al.*, 2008; Olmstead *et al.*, 2013). Chloride-based salts have traditionally been the focus of freshwater salinization, but recent studies suggest salinization results from a complex mixture of ions (e.g. Cl^−^, Na^+^, Ca^2+^, Mg^2+^ and K^+^; Kefford *et al.*, 2002, 2016; Griffith, 2014; Kaushal *et al.*, 2018), requiring more research to evaluate the effects of non-chloride salts at different concentrations (Cañedo-Argüelles *et al.*, 2013; Iglesias, 2020). Sodium bicarbonate (NaHCO_3_^−^) is a major constituent of ONGD produced waters and increased Na^+^ and HCO_3_^−^ ion concentrations have been associated with ONGD in several freshwater ecosystems (Patz *et al.*, 2004; Farag and Harper, 2014; Harper *et al.*, 2014), including streams in this study (Walker and Walters, 2019a; Walters *et al.*, 2019). Additionally, ONGD can alter water temperatures indirectly through decreased vegetative cover and increased solar radiation or directly via increased produced water discharge (Davis *et al.*, 2009). Thus, ONGD-related shifts in NaHCO_3_^−^ concentrations and temperatures could negatively affect the physiology of freshwater organisms (Harper *et al.*, 2014).

**Figure 1:**
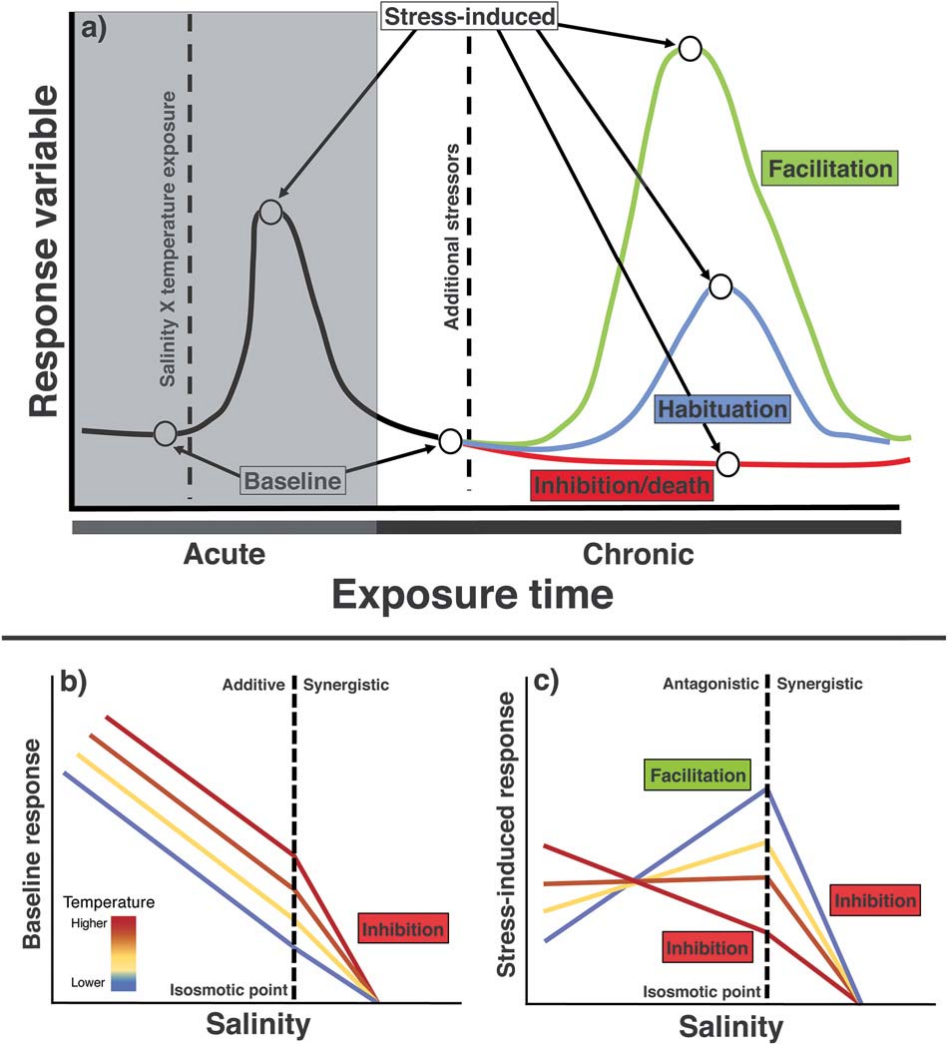
Conceptual diagram depicting potential hormone responses (i.e. facilitation, acclimation and inhibition) following chronic exposure to multiple stressors (i.e. salinity and temperature) and acute exposure to additional stressors [(a) i.e. capture, handling, blood sampling and confinement]. Predicted relationships for how increased freshwater salinization and temperatures would affect baseline (b) and stress-induced (c) responses in freshwater, stenohaline fishes. Stress-induced concentrations and the difference between baseline and stress-induced concentrations (i.e. absolute change) were used to represent an individual’s ability to mount a stress response (i.e. stress reactivity). Baseline samples were taken within 3 minutes of capture, while stress-induced samples were taken from the same individual approximately 30 minutes after the baseline samples. The shaded area reflects the prior experience of fish, which was not measured in this study. Diagram adapted from Herman (2013).

Animals experience and respond to stress in myriad ways, wherein allostatic mediators modulate physiological condition for maintaining homeostasis (Fig. 1a; Barton, 2002; Romero, 2004). Primary allostatic mediators, such as glucocorticoid hormones (e.g. cortisol), are released to divert energetic investment (e.g. glucose) for the duration of stressors. If individuals are chronically exposed to an initial stressor, hormone regulation [e.g. hypothalamus–pituitary–interrenal axis (HPI) in fishes] and the capacity to mount a physiological response (i.e. stress reactivity) to novel stressors is modified (Sapolsky *et al.*, 2000; Romero, 2004; Herman, 2013). Individuals unable to mount appropriate responses will likely exhibit a diminished response where they fail to increase circulating hormones when exposed to additional stressors, resulting in an inhibitory response and potentially death (i.e. inhibition; Fig. 1a; Herman, 2013). But some individuals may survive chronic exposure to the initial stressors, resulting in an adaptive response where hormone responses are amended and survival is increased (Cyr and Romero, 2007; Herman, 2013). These adaptive responses include dampened (i.e. acclimation) or heightened (i.e. facilitation) stress reactivity, where individuals reduce or elevate energy-mobilizing hormones when presented with additional stressors, respectively (Fig. 1a; Herman, 2013). Yet, the extent to which these responses emerge following chronic exposure to multiple stressors is essentially unknown, restricting our ability to predict population responses to future stressors (Todgham and Stillman, 2013).

Every species has a range of optimal conditions for physiological performance, which declines outside of those conditions (Huey and Stevenson, 1979; Angilletta *et al.*, 2002). Thus, increased salinization and water temperature will affect freshwater organisms through changes in ion regulation, acid/base balance, circulating hormones and metabolic pathways (Mazik *et al.*, 1991; Morgan and Iwama, 1998; Evans, 2008; Hopkins *et al.*, 2016). The conventional theory of osmoregulation predicts that as salinity increases towards a species’ isosmotic point (i.e. an internal osmolality that is equal to the external environment), freshwater organisms should allocate less energy towards ion regulation and water exchange (Kefford, 2019). Glucocorticoids are important energy-mobilizing hormones, and so baseline cortisol and glucose concentrations and metabolic activity should correspondingly decline with increased salinity, until the species’ isosmotic threshold is reached (i.e. ~9 ppt; Peterson and Meador, 1994; Boeuf and Payan, 2001). At which point, osmoregulation and metabolic activity for individuals exposed to higher salt concentrations becomes energetically costly (Boeuf and Payan, 2001). In fact, many studies show that cortisol concentrations increase in saltwater fishes transferred to freshwater, while concentrations usually decline in freshwater fishes exposed to higher salinities (McCormick, 2001). But other studies indicate that chronic exposure to salinities >0.5 ppt often results in negative sub-lethal effects for many freshwater organisms (Peterson and Meador, 1994; Farag and Harper, 2014; Kültz, 2017). In addition, rising temperatures will generate greater thermal stress further challenging fish physiologically (Davis and Parker, 1990; Pörtner and Farrell, 2008; Kaushal *et al.*, 2010; Buckley *et al.*, 2012). Indeed, several studies have documented increased cortisol and glucose concentrations (Davis, 2004; Meka and McCormick, 2005), as well as other physiological changes for fishes at higher temperatures (Lermen *et al.*, 2004).

Given the individual effects of these stressors on circulating hormones and knowing that temperature has a strong influence on chemical toxicity (Wang *et al.*, 2019), we expect salinity and temperature to interact in complex ways. Here, our main objective was to evaluate the individual and interactive effects of salinity and temperature on the physiological responses—cortisol and glucose—of three freshwater, stenohaline fishes. Our second objective was to evaluate a fish’s ability to mount a physiological response when exposed to additional stressors (i.e. stress reactivity; Table S1). We combined field and laboratory studies using age 1+ Mottled sculpin (hereafter Sculpin; *Cottus bairdii*), Mountain Sucker (Sucker; *Catostomus platyrhynchus*) and Colorado River Cut-throat Trout (Trout; *Oncorhynchus clarki pleuriticus*). We hypothesized that salinity and temperature would have a strong influence on circulating cortisol and glucose concentrations, whereby the effect of salinity would be temperature dependent. Based on osmoregulation and metabolic theory, we predicted that

(1) baseline cortisol and glucose would decline as salinity increased but would increase positively with temperature, representing individual stressor effects or additive relationships between salinity and temperature (i.e. the net effect is equal to the sum of individual effects). For example, baseline cortisol and glucose concentrations should be greater at higher temperatures due to greater metabolic demands (Brown *et al.*, 2004) and decrease as temperatures decline and salinities increase, reflecting a reduction in the energy needed for osmoregulation and metabolism (Fig. 1b);

(2) a reduction or limited response in baseline concentrations could promote a heightened stress reactivity when exposed to additional stressors (i.e. facilitation), having greater stress-induced and absolute change in concentrations at higher salinities and lower temperatures, weakening as temperatures increase to reflect antagonistic interactions (i.e. net effect less than the sum of individual effects) between salinity and temperature (i.e. inhibition; Fig. 1c);

(3) however, if salinities or temperatures surpass the species’ salinity and temperature thresholds, these stressors would interact synergistically (i.e. the net effect is greater than the sum of individual effects), resulting in substantially reduced concentrations or death at higher temperatures and salinities (i.e. inhibition; Fig. 1b and c).

## Materials and methods

### Field study

#### Study streams

We conducted our field study in headwater streams along the Wyoming Range in the Upper Green River basin, Wyoming, USA (Table 1; Fig. S1). Many of the streams are within the LaBarge oil and gas field, where ONGD has occurred since the early 1900s and continues to expand. Previous studies from the Upper Green River, Powder River and Tongue River basins in Wyoming have linked increased land-use change associated with ONGD to elevated concentrations of Na^+^, HCO_3_^−^, Cl^−^, SO_4_^2−^, Ca^2+^ and Mg^2+^ ions in local streams (Patz *et al.*, 2004; Farag and Harper, 2014; Harper *et al.*, 2014; Walters *et al.*, 2019).

**Table 1:** List of stream locations and species sampled in 2015, 2016 and 2017 to evaluate the individual and interactive effects of salinity and temperature on fish physiology in the Wyoming Range of the Upper Green River basin, Wyoming

Sub-drainage	Stream	Latitude	Longitude	Well density (no. km^−2^)	Sample year	Species sampled	Temperature	Salinity
Dry Piney	Dry Piney	42.3861	−110.2761	2.57	2015, 2016, 2017	MTS	13.0, 11.0, 14.7	591, 666, 633
Dry Piney	Black Canyon	42.3673	−110.3161	1.06	2015, 2016	MSC, MTS	11.5, 11.2	563, 630
Dry Piney	Fogarty 1	42.3879	−110.2705	2.78	2015, 2016	MTS	13.0, 11.8	487, 433
Dry Piney	Fogarty 2	42.4103	−110.3395	1.05	2015, 2016	MSC	12.7, 11.8	475, 433
South Piney	South Beaver	42.4546	−110.3807	0.30	2015, 2016	MSC, MTS	14.3, 9.3	431, 419
South Piney	North Beaver	42.4661	−110.3803	0.29	2015, 2016	MSC, MTS	13.9, 11.7	461, 413
South Piney	Middle Beaver	42.4607	−110.3945	0.34	2015, 2016	MSC, MTS	12.1, 8.5	443, 406
South Piney	Fish	42.5518	−110.4585	0.00	2015, 2016, 2017	MSC	5.9, 10.0, 6.1	327, 310, 304
Cottonwood	South Cottonwood	42.8470	−110.3919	0.00	2016, 2017	MSC	10.1, 5.9	399, 401
Cottonwood	North Cottonwood	42.8741	−110.3642	0.00	2016, 2017	MSC, MTS	14.5, 13.2	342, 364
Horse	Horse	42.9318	−110.4672	0.00	2017	MSC, MTS	8.7	340
South Beaver	Buck	42.9848	−110.4062	0.00	2017	MTS	13.5	447
South Beaver	South Beaver: Rim	42.9916	−110.4046	0.00	2017	MSC, MTS	15.1	377
South Beaver	Chall	43.0200	−110.3979	0.00	2017	MTS	9.8	403

Sample streams are listed in order from south to north, which also corresponds to a gradient of ONGD within the basin. Temperature and salinity values represent *in situ* measurements from each year, respectively.

*Notes*: Salinity was measured as specific conductivity (μS_25°C_ cm^−1^) and temperature was measured in °C. MSC = mottled sculpin; MTS = mountain sucker. Catchment percent land-use change followed the same pattern as well density but are not reported here (range reported in text). Study map can be found in Fig. S1.

All study streams originate from springs on the eastern side of the Wyoming Range. Our study sites overlap those described in Walters *et al.* (2019), where catchment land-use change associated with ONGD ranged from 0.0% to 9.2% and well density ranged from 0.0 to 2.8 wells km^−2^ (Table 1). In the farthest south in the Dry Piney drainage, we sampled fish from Dry Piney, Black Canyon and Fogarty Creek, which flow through more intense development (3.2–9.2%). Moving north, we sampled the less developed South Piney drainage (1.0–2.3%), which includes South Beaver, Middle Beaver, North Beaver and Fish Creeks. The remaining reference streams (i.e. Fish, South Cottonwood, North Cottonwood, Horse, Buck, South Beaver: Rim and Chalk creeks) further north are within the Bridger–Teton National Forest and have little to no ONGD in the drainages (Table 1; Fig. S1).

Discharge in the Upper Green River basin is dominated by snowmelt in early spring, with rainfall controlling fluctuations in baseflow during the rest of the year. Riparian corridors in the basin are mostly narrow, dominated by willows with aspen patchily distributed at higher elevations. Upland vegetation throughout the basin is mostly drought-tolerant sagebrush and conifers at higher elevations. And all drainages have comparable levels of cattle grazing (Walters *et al.*, 2019), so we would expect the effect of cattle grazing on salinity to be similar between watersheds (Waldner *et al.*, 2001).

#### Water quality

Prior to sampling each site in each year, we measured *in situ* water temperature (°C) and salinity [i.e. specific conductivity (μS_25°C_ cm^−1^)] at the most downstream location in each stream using a handheld YSI Professional plus metre (YSI Inc., Yellow Springs, OH). The time between *in situ* measurements and fish sampling never exceeded 4 hours and no major changes in temperature, precipitation or flows that occurred during sampling to affect *in situ* measurements. *In situ* measurements were monitored multiple times (8–20) in the summer of 2016 and 2017 and are reflective of differences in water quality among sample streams during stable baseflow conditions (Walker and Walters, 2019a; Walters *et al.*, 2019). We used *in situ* temperature and salinity as predictors in all field analyses because they were most representative of baseline conditions experienced by fish. *In situ* salinity and temperature ranged from 304–666 μS_25°C_ cm^−1^ and 5.9–15.1°C across all sites and years in the field study (Table 1; Fig. S2).

#### Fish sampling and processing

We measured physiological responses of Sculpin and Sucker in the field across a gradient of salinity and temperature in 8–10 headwater streams in August 2015–2017 (Table 1; Fig. S1). Sampling occurred in August to ensure that physiological responses were not influenced by reproductive status (Romero, 2002), which is between April and June for both species. We focused our field study on these two species because they are the dominant fishes in many streams of this region (Dauwalter, 2013). In addition, Sculpin are widespread throughout North America (Petty and Grossman, 1996), while Sucker has a broad distribution throughout the western USA and Canada (Scott and Crossman, 1973). We were unable to measure the physiological responses of Trout in the field due to their limited distribution across the ONGD gradient and status as a species of conservation concern (Hirsch *et al.*, 2013).

Each year, we collected several (5–40 individuals) age 1+ of Sculpin (55–132 mm) and Sucker (60–175 mm) from streams using backpack electrofishing (Smith-Root LR-24, Vancouver, WA, USA; Table 1). Immediately after capture, we briefly placed individual fish in a solution of MS-222 (0.29 g L^−1^) to be lightly anaesthetized for safe blood extraction. Within 3 minutes of capture, we collected baseline blood samples via the caudal vein with a 27-gauge, heparinized syringe (Lawrence *et al.*, 2018). After we extracted the baseline blood samples, we measured standard length (1 mm) and mass (0.01 g) from all individuals and placed them into flow-through chambers in each stream. In 2016 and 2017, we collected a stress-induced blood sample 30–35 minutes after baseline sampling for all individuals to measure stress reactivity related to capture, handling, blood extraction and confinement. While variable among species, several studies show cortisol levels for many fishes peak 30–45 minutes after initial stress exposure (Barton and Iwama, 1991; Jentoft *et al.*, 2005). Fish were anaesthetized a second time before collecting the stress-induced sample. All fishes were retained in the flow through chambers under natural stream conditions, monitored for mortality over 48 hours and released into their respective stream. The mortality associated with processing and blood extraction was low in all years, never exceeding 10%. All animals were handled according to the University of Wyoming Institutional Animal Care and Use Committee protocol #20150610AW00171-02.

### Acute and chronic, multiple-stressor experiments

#### Source of fish

Because numerous other factors could influence fish responses in the field, we conducted laboratory experiments that manipulated temperature and salinity to evaluate the physiological responses of Sculpin, Sucker and Trout. In August 2017, we collected Sculpin and Sucker from five and seven streams, respectively (Table 1). We also received 150, 10-cm hatchery-raised Trout from the Wyoming Game and Fish Department’s Fish Hatchery near Daniel, Wyoming, in April 2018. All fish were transported in a 284-L aerated tank at 10°C to the University of Wyoming’s Red Buttes Environmental Biology Laboratory in Laramie, Wyoming. We held fish in continuous flow through aquaria at 10°C for at least 2 months prior to experimentation to allow acclimation to laboratory conditions. One month prior to experimentation, temperatures were slowly adjusted (0.5°C per day), so that fish could acclimate to experimental temperature treatments (see below). During the acclimation period and experiments, fish were maintained under a natural photoperiod of 14:10 for August in Wyoming and each fish was fed approximately 0.5 g of bloodworms daily (Brine Shrimp Direct, Inc., Ogden, Utah).

#### Experimental setup

We manipulated temperature (two levels) and salinity (six levels) using a modified, continuous-flow mini-diluter system (Benoit *et al.*, 1982), resulting in 12 different temperature–salinity treatments. With the diluter system, we conducted 3-day acute (Trout only) and 32-day chronic (Trout, Sculpin and Sucker) temperature–salinity experiments for each species separately. The diluter system included (i) two large head tanks where target water temperatures were manipulated and maintained, (ii) one diluter chamber for each temperature treatment where sodium bicarbonate (NaHCO_3_) was pumped in from a stock solution of 95 g L^−1^ to generate six salinity treatments starting at 1400 μS_25°C_ cm^−1^ with a 0.75-fold dilution series and (iii) six splitter boxes that separated into four replicate aquaria (n = 48). The flow rate into each aquarium was 0.125 L min^−1^, resulting in approximately three water changes per day. We measured conductivity, dissolved oxygen (% and mg L^−1^), and pH in each aquarium daily using a handheld YSI Professional plus metre.

Temperature treatments included a lower temperature of 14–16°C, representing an optimal thermal temperature for these species (Hasnain *et al.*, 2010), and a higher temperature of 18–22°C, representing temperatures closer to each species’ maximum thermal tolerances (Walters *et al.*, 2018; Mandeville *et al.*, 2019). Due to variable ambient laboratory conditions and because fish have acute sensitivity to small temperature changes, we monitored and measured temperatures hourly in each aquarium using HOBO temperature loggers (Onset Computer Corporation, Bourne, Massachusetts, USA). Over the course of each experiment, we maintained temperatures within a narrow range of target temperatures (Fig. S3). There was a wider range of temperatures in the Trout experiments because we refined temperatures at the start of the chronic Trout experiment. We reduced the highest temperature to 18°C for the chronic Trout experiment, as most individuals died at 22°C before salt was added. We used mean temperature for each aquarium from each experiment as a continuous predictor variable in our models. For each experiment, we successfully maintained temperatures near the target temperatures, which were significantly different between temperature treatments: Trout (14.15 ± 0.45°C and 18.27 ± 0.21°C; t = −49.4, df = 28.6, *P* < 0.0001), Sculpin (16.63 ± 0.53°C and 21.46 ± 0.19°C; t = −41.8, df = 29.1, *P* < 0.0001) and Sucker (15.88 ± 0.49°C and 21.69 ± 0.12°C; t = −55.0, df = 25.8, *P* < 0.0001; Fig. S3).

We selected salinity ranges that reflected measurements reported from field surveys between 2012 and 2018 (Walters *et al.*, 2019) and represent ranges used in other NaHCO_3_ studies (Farag and Harper, 2014; Harper *et al.*, 2014). The salinity gradient included six different treatments: a control treatment where no NaHCO_3_ was added (370 μS_25°C_ cm^−1^) and five NaHCO_3_ treatments to achieve a salinity gradient from approximately 400 to 1400 μS_25°C_ cm^−1^ (0.2–0.7 ppt; Fig. S4). Overall, we maintained stable laboratory conditions for the desired temperature–salinity treatments for each experiment (Fig. S5).

We randomly assigned one fish among 48 aquaria (20 L; 76.2 × 30.5 × 30.5 cm). Before placing fish into aquaria, we measured standard length (1 mm) and mass (0.01 g) of all individuals to monitor growth in the chronic experiments. All aquaria were continuously aerated throughout the experiments and were cleaned and maintained daily. We monitored fish daily for fungal growth and mortality. If fish exhibited signs or death due to fungal growth, they were replaced with new, uninfected individuals. The new individuals were maintained for a full 32-day experiment. We made qualitative observations of food consumption but were unable to evaluate food consumption quantitatively. At the end of each experiment, we removed fish from aquaria and lightly anaesthetized them in a MS-222 (0.29 g L^−1^) solution. We extracted blood samples with a 27-gauge, heparinized syringe via the caudal vein within 3 minutes of netting and measured the final length and mass of each fish. Fish were placed back into their respective aquaria where a stress-induced blood sample was collected 30–35 minutes later.

### Blood processing

For the field study and laboratory experiments, baseline and stress-induced glucose were measured in 0.6 μL of whole blood using an ACCU-CHECK Aviva glucose metre immediately following blood extraction (Roche Diabetes Care, Inc., Indianapolis, Indiana). Blood samples were placed on ice for further processing (<5 hours), at which time plasma was separated from red blood cells via centrifuging for 10 minutes. All plasma samples were then frozen and stored at −20°C until further laboratory assays were conducted.

Baseline and stress-induced cortisol concentrations were measured in a validated volume of blood plasma using a modified protocol (Moore, 1986; Neuman-Lee and French, 2017). Briefly, extractions were performed using a solution of 30% ethyl acetate–isooctane. Samples were resuspended using phosphate-buffered saline solution and assayed in duplicate for cortisol (Fitzgerald 20-CR45, Lot #P0012502). Individual recoveries were determined using a separate aliquot of the resuspended fractions. Recoveries are used to account for any potential loss of hormone during extractions and to correct final sample concentration. The average intra-assay coefficient of variation (CV) was 19.1% and the inter-assay CV was 9.6%.

### Statistical analyses

Fish survival analyses for the laboratory experiment only included mortalities associated with the temperature–salinity treatments and exclude all mortalities resulting from fungal activity. We excluded mortalities from all other models, as they did not contribute to the remaining physiological responses. Absolute change in cortisol and glucose were calculated as the absolute difference between baseline and stress-induced concentrations for individuals. We used stress-induced and absolute changes in concentrations from baseline as measures of stress reactivity in this study. We calculated Fulton’s condition factor (K = (*W* × 10^5^)/*L*^3^) to evaluate the relationship between predictor variables and condition for each species across streams in the field and treatments in the laboratory experiments, where *W* represents fish mass and *L* represents fish standard length. For the chronic, multiple-stressor experiments, we calculated instantaneous growth rates for individual fish as the change in fish wet weight and standard length from the beginning and the end of the experiment.

For the field study and laboratory experiments, we evaluated the individual and interactive effects of salinity and temperature on the physiological responses of fishes using a mixed-effect modelling approach with a gamma distribution (continuous positive data). We conducted initial data exploration steps that included examination of response metric distributions and collinearity between predictors (Zuur *et al.*, 2010). To account for the sampling of multiple individuals from each stream and multiple years in the field study, we included a random effect of stream nested within sample year. To account for multiple replicates per treatment in the laboratory experiments, we included a random effect of the temperature–salinity treatment in each model. For all models, we used non-overlapping 95% confidence limits and *P*-values to assess the importance of fixed effects in all models (Nakagawa and Cuthill, 2007). For all models, we used a two-step approach to evaluate the relationships between salinity and temperature on the 48 response metrics evaluated in this study (Table 2). First, we evaluated the interactive effect of salinity and temperature for each response metric. If the 95% confidence limit for the interaction term overlapped zero and the *P*-values were >0.05, the interaction term was removed from the model. In Step 2, we evaluated the relationships between salinity and temperature using an additive model using the above confidence limit and *P*-values criteria. We then visually inspected model assumptions using plots of fitted and residual values and evaluated overdispersion (i.e. greater variability in the data than expected). We centred and rescaled the salinity using the ‘scale’ function to improve model convergence. All analyses were performed in Program R (R version 3.5.1; R Core Team, 2018) with R package ‘lme4’ (Bates *et al.*, 2015).

**Table 2:** Summary of the mixed-effects models evaluating the individual and interactive effects of salinity and temperature on the physiological responses of Trout, Sculpin and Sucker from field study and from the acute (3-day) and chronic (32-day) laboratory experiments

Response metric	Sculpin Field survey	Sucker Field survey	Trout Acute experiment	Trout Chronic experiment	Sculpin Chronic experiment	Sucker Chronic experiment
Baseline						
Cortisol	Additive (−,−)	Additive (−,+)	Temperature (−)	NS	NS	Antagonistic (−,+)
Glucose	NS	NS	NS	NS	NS	NS
Stress-induced						
Cortisol	NS	NS	NS	NS	NS	Temperature (−)
Glucose	Additive (+,+)	NS	Antagonistic (+,-)	NS	NS	NS
Absolute change						
Cortisol	Antagonistic (+,+)	Antagonistic (+,+)	Temperature (+)	NS	Temperature (−)	Temperature (−)
Glucose	Salinity (+)	NS	NS	NS	NS	NS
Survival	NA	NA	NA	Temperature (−)	Temperature (−)	Temperature (−)
Instantaneous growth rate	NA	NA	NA	NS	NS	NS
Condition	NS	NS	NS	NS	NS	NS

+ and − symbols in parentheses represent the direction for each corresponding relationship. When additive and antagonistic relationships are reported, symbols in parentheses are in order of the overall effect of salinity and temperature, respectively.

*Notes*: NA represents variables that were not measured and NS represents insignificant relationships. Salinit y was measured as specific conductivity (μS_25°C_ cm^−1^) and temperature was measured in °C. Baseline samples were collected within 3 minutes of capture and stress-induced samples were collected 30 minutes after baseline samples. Absolute change in cortisol and glucose were calculated as the change in concentrations from the baseline and stress-induced samples.

## Results

### Field study (Sculpin, Sucker)

Of the 14 response metrics evaluated in the field study, 8 were unrelated to *in situ* salinity and temperature (Tables 2 and S2). The remaining six relationships were best explained by salinity in isolation (one metric); simple, additive relationships (three metrics); and complex antagonistic interactions (two metrics) between salinity and temperature (Tables 2 and S2).

#### Condition

Body condition did not vary with salinity or temperature for either species in the field (Tables 2 and S2).

#### Baseline physiology

**Figure 2:**
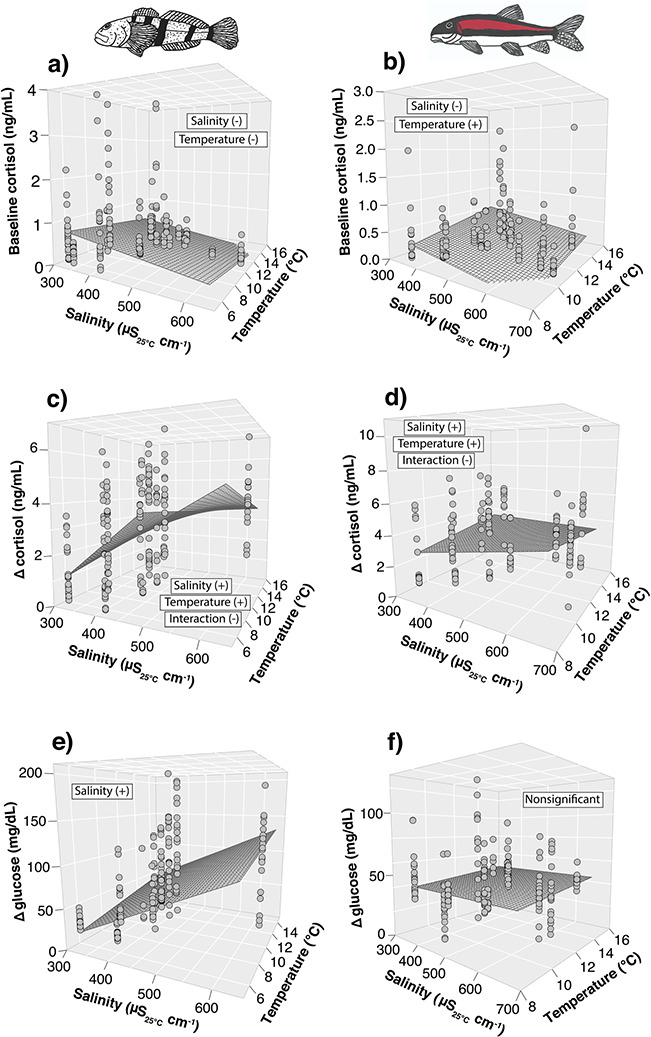
Representative relationships for mixed-effect models evaluating the individual and interactive effects of salinity and temperature on mottled sculpin (*Cottus bairdii*; first column) and mountain sucker (*Catostomus platyrhynchus*; second column) physiology in the field in 2015, 2016 and 2017. Shown are the predicted 3D response surfaces for baseline cortisol (a and b), absolute (Δ) in cortisol (c and d), absolute change (Δ) in glucose (e and f) for each species. Δ in glucose followed a similar pattern as stress-induced glucose for Sculpin (not shown). All models included a random effect of stream nested within a year. Baseline samples were taken within 3 minutes of capture and stress-induced samples were taken 30 minutes after the baseline sample. Stress-induced samples were not taken in 2015. Absolute change was calculated as the change in concentration between baseline and stress-induced samples. Terms in boxes represent the model results for each relationship followed by the overall effect of salinity, temperature and their interaction in parentheses.

Basal cortisol concentrations declined as salinity increased for both species (Tables 2 and S2); however, temperature effects differed between species with concentrations decreasing for Sculpin (Fig. 2a) and increasing for Sucker (Fig. 2b) as temperatures increased. Basal glucose concentrations did not vary with salinity or temperature for either species (Tables 2 and S2).

#### Stress-induced physiology

Stress-induced cortisol concentrations did not vary with salinity or temperature for either species (Tables 2 and S2). Absolute change in cortisol increased with salinity at lower temperatures, but the effect of salinity declined as temperature increased for both species, reflecting antagonistic interactions (Tables 2 and S2; Fig. 2c and d). The stress-induced glucose increased additively with salinity and temperature, while the absolute change in glucose was solely driven by a positive relationship with salinity for Sculpin (Tables 2 and S2; Fig. 2e). Neither parameter was related to stress-induced or change in glucose for Sucker (Tables 2 and S2; Fig. 2f).

### Laboratory experiments (Trout, Sculpin, Sucker)

Across all response metrics evaluated in the laboratory experiments (34 metrics), 24 were unrelated to the temperature–salinity treatments (Tables 2, S3 and S4). The 10 significant relationships were best explained by the effect of temperature in isolation (eight metrics), usually being negative relationships, and antagonistic interactions (two metrics) between salinity and temperature (Tables 2, S3 and S4).

### Acute, multiple-stressor experiment (Trout)

#### Survival and condition

All Trout survived in the acute, multiple-stressor experiment. Fish condition did not differ among the temperature–salinity treatments (Tables 2 and S3).

#### Baseline physiology

**Figure 3:**
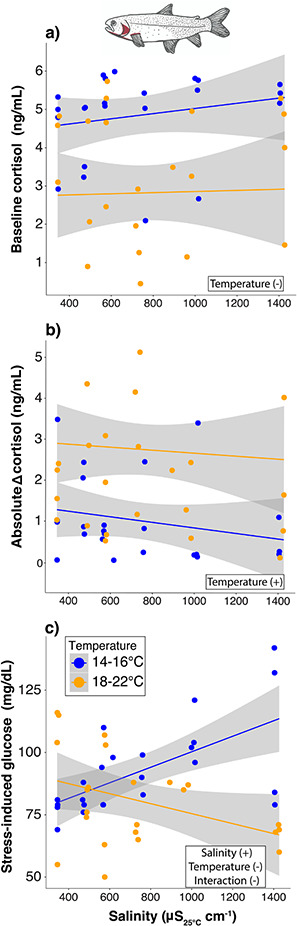
Relationships showing the mixed-effect model results evaluating the individual and interactive effects of salinity and temperature on Colorado River cutthroat trout (*Oncorhynchus clarki*) physiology from the acute (3-day) multiple-stressor experiment. Shown are the predicted responses for baseline cortisol (a), absolute change (Δ) in cortisol (b) and stress-induced glucose (c) for Trout. All models included a random effect of individual nested within treatment. Baseline samples were taken within 3 minutes of capture and stress-induced samples were taken 30 minutes after the baseline sample. Absolute change was calculated as the change in concentration between baseline and stress-induced samples. Terms in boxes represent the model results for each relationship followed by the overall effect of salinity, temperature and their interaction in parentheses.

Basal cortisol concentrations negatively corresponded with temperature for Trout (Tables 2 and S3; Fig. 3a). Basal glucose concentrations did not vary with either salinity or temperature (Tables 2 and S3).

#### Stress-induced physiology

Stress-induced cortisol concentrations did not vary with either salinity or temperature for Trout (Tables 2 and S3). Absolute change in cortisol concentrations was positively related to temperature (Tables 2 and S3; Fig. 3b). Stress-induced glucose concentrations increased with salinity at lower temperatures but declined with salinity at higher temperatures, representing an antagonistic interaction (Tables 2 and S3; Fig. 3c). Absolute change in glucose concentrations did not vary with either salinity or temperature (Tables 2 and S3).

### Chronic, multiple-stressor experiments (Trout, Sculpin, Sucker)

#### Survival, growth and condition

**Figure 4:**
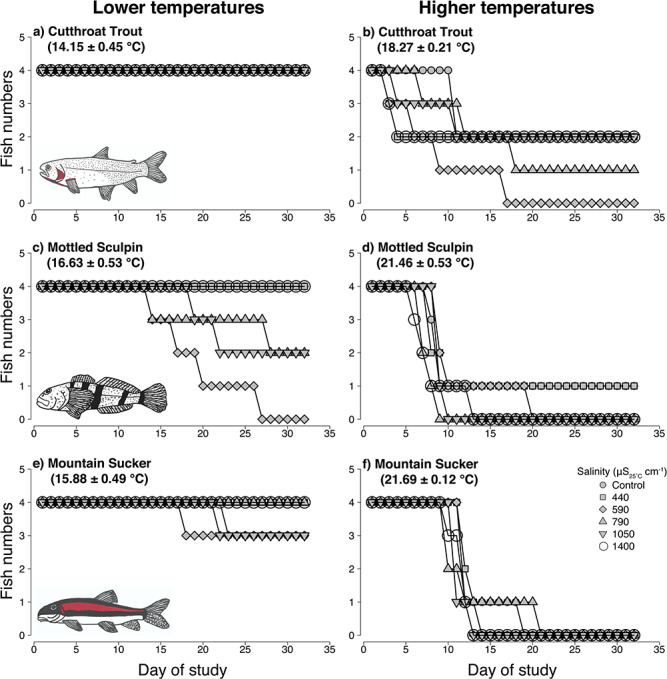
Data represents survival of Colorado River cutthroat trout (a and b; CRCT), mottled sculpin (c and d) and mountain sucker (e and f) during the chronic (32-day) multiple-stressor experiments. Compared with the lower temperature treatments, higher temperatures were negatively related to survival for all species. Salinity was unrelated to survival for all species. All CRCT survived the acute (3-day) multiple-stressor experiment (not shown). The left column represents survival in lower temperature treatments and the right column represents survival at higher temperature treatments. Values in parentheses represent the mean ± SD for each experiment. Each target salinity treatment reflects the four replicate aquaria. Data for each species represents a gradient of 12 different temperature and salt treatment combinations.

Survival negatively corresponded with temperature for all species in the chronic experiments (Tables 2, S3 and S4). Most individuals from the lower temperature treatment survived to the end of the study (Fig. 4). In the higher temperature treatment, no Suckers, two Sculpin and nine Trout survived to the end of the study (Fig. 4). Instantaneous growth rate and condition did not vary with salinity or temperature for any species (Tables 2, S3 and S4).

#### Baseline physiology

**Figure 5:**
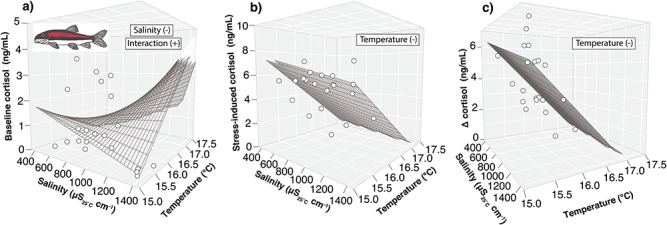
Shown are the predicted 3D response surfaces for baseline (a), stress-induced (b) and absolute change (Δ) in cortisol (c) for mountain sucker (*Catostomus platyrhynchus*) from the mixed-effects models evaluating the individual and interactive effects of salinity and temperature in the chronic (32-day) multiple-stressor experiments. Absolute change in cortisol for Sculpin followed a similar pattern as Sucker (not shown). Baseline samples were taken within 3 minutes of capture and stress-induced samples were taken 30 minutes after the baseline sample. Absolute change was calculated as the change in concentration between baseline and stress-induced samples. Terms in boxes represent the model results for each relationship followed by the overall effect of salinity, temperature and their interaction in parentheses.

Basal cortisol concentrations did not vary with salinity or temperature for Sculpin and Trout (Tables 2, S3 and S4) but decreased with salinity at lower temperatures and increased with salinity at higher temperatures for Sucker (Tables 2 and S4; Fig. 5a). Basal glucose concentrations did not vary with salinity or temperature for any species (Tables 2,  S3 and S4).

#### Stress-induced physiology

Stress-induced cortisol concentrations did not vary with salinity or temperature for Sculpin and Trout (Tables 2, S3 and S4), but concentrations declined with temperature for Sucker (Tables 2 and S4; Fig. 5b). Absolute change in cortisol did not vary with salinity or temperature for Trout (Tables 2 and S3) but negatively corresponded with temperature for Sculpin and Sucker (Tables 2 and S4; Fig. 5c). Stress-induced and absolute change in glucose concentrations did not vary with salinity or temperature for any species (Tables 2, S3 and S4).

## Discussion

We combined field study and laboratory experiments to evaluate the individual and interactive effects of increased freshwater salinization and temperature on the physiological responses of three freshwater, stenohaline fishes. Our laboratory experiments complement our field study, as salinities and temperatures ranged from 304 to 666 (μS_25°C_ cm^−1^) and 5.9 to 15.1 (°C) in the field and 400 to 1400 (μS_25°C_ cm^−1^) and 14–22°C in the laboratory experiment, respectively. Overall, our results demonstrate that (i) temperature and salinity are important physiological mediators for freshwater fishes, (ii) the effect of salinity is temperature dependent and (iii) chronic exposure to higher salinities can facilitate stronger stress reactivity responses to additional stressors, which weakens as temperatures increase.

### Baseline physiology

Our first prediction that baseline cortisol and glucose concentrations would decline with salinity and temperature was partially supported. Across all studies and species, baseline concentrations were either unaffected by or declined across the salinity gradient with the effect of temperature differing between species. We offer two hypotheses to potentially explain the observed pattern of reduced baseline concentrations at higher salinities. First, individuals may have been chronically stressed by increased salinity or other stressors, whereby surviving individuals downregulated the release of cortisol via a negative feedback loop in the HPI axis to avert deleterious effects of persistently elevated cortisol levels (Romero, 2004; Rich and Romero, 2005; Cyr and Romero, 2007). Second, the conventional theory of osmoregulation states that as salinity increases, less energy should be allocated towards osmoregulation, reflecting a reduction in baseline energy-mobilizing hormones and associated glucose levels (Jentoft *et al.*, 2005; Kefford, 2019). Our findings likely support the latter hypothesis and previous research suggesting increased salinity can provide beneficial effects to freshwater organisms, as long as salinities remain below the species’ isosmotic point (Boeuf and Payan, 2001; Kefford, 2019). Given the physiological responses to salinity in our study, it is apparent that these fishes could acclimate to the salinity gradient and that salinities remained below the isosmotic threshold, which has been observed at ~9 ppt for many freshwater fishes (Peterson and Meador, 1994). Regardless of the mechanism, reductions in the energetic costs of osmoregulation with increased salinity potentiate shifts in investment towards other physiological processes, such as growth, reproduction or mounting a response to additional stressors.

Based on metabolic theory, we also predicted that baseline concentrations would increase with temperature due to greater metabolic demand (Brown *et al.*, 2004). This was only partially supported and was species dependent. Baseline concentrations did not differ or declined with temperature for species more frequently found at less degraded sites with lower average temperatures (i.e. Trout and Sculpin) and increased positively for species found at more degraded sites with higher temperatures (i.e. Sucker; Dauwalter, 2013; Walters *et al.*, 2018; Mandeville *et al.*, 2019). Such differences in baseline concentrations associated with temperature have been linked to distinctions in cellular biochemistry and regulatory processes between species, such as evolutionary differences in the mechanisms driving mitochondrial bioenergetics and biogenesis (Johnston *et al.*, 1994; Bremer and Moyes, 2011).

### Stress-induced physiology

Determining the environmental conditions under which individuals, chronically exposed to multiple-stressors, can mount a physiological response (i.e. stress reactivity) to novel stressors is important for conservation. We evaluated an individual’s ability to mount a physiological response when chronically exposed to elevated salinities and temperatures. In accordance with our second prediction, we found several instances where a reduction in baseline cortisol and glucose concentrations at higher salinities and lower temperatures facilitated stronger stress reactivity responses (i.e. greater stress-induced and absolute change in cortisol and glucose) to additional stressors. But this facilitative response weakened as temperatures approached and surpassed the species’ optimal–thermal point (Hasnain *et al.*, 2010), representing antagonistic interactions between salinity and temperature. These antagonistic interactions may reflect the species’ co-tolerance to the temperature–salinity ranges we evaluated, where sensitivity to one stressor increased resistance to another stressor (Vinebrooke *et al.*, 2004; Côté *et al.*, 2016). Although facilitative responses to chronic stress have been demonstrated under controlled conditions (Dallman *et al.*, 1992; Marti *et al.*, 1994; Bhatnagar and Vining, 2003), the responses of wild, free-living animals remain an important area to explore (Romero, 2004; Dickens and Romero, 2013; Dantzer *et al.*, 2014). While seemingly rare, facilitation of the HPI axis to chronic stressors can be advantageous for maintaining responsiveness to acute stressors, but this ultimately depends on the type and magnitude of each stressor (Dallman and Bhatnagar, 2001), along with their interactive effects (Jackson *et al.*, 2016).

The greatest differences in stress-induced responses we observed were between the field survey and chronic experiments but were similar for species experiencing the same conditions. For example, stress-induced responses were driven by an antagonistic interaction between salinity and temperature for Sculpin and Sucker in the field survey, as we predicted, but were negatively related to temperature in the laboratory experiments for both species. The differing stress-induced responses between the field and laboratory studies are likely an artefact of different temperature ranges with field temperatures (5.9–15.1°C) remaining below and laboratory temperatures being near and above (14–22°C) the species’ thermal optima (~16°C; Hasnain *et al.*, 2010). Combined with lower survival for all species and the negative stress-induced relationships observed in our laboratory experiments, our results suggest that chronic exposure to temperatures closer to species’ critical thermal maxima can lead to physiological inhibition and eventually death, regardless of elevated salinity.

### Synergistic interactions

Our third prediction was that salinity and temperature would interact synergistically to affect baseline and stress-induced physiological responses, but only in cases where both stressors were above the species’ isosmotic and optimal–thermal points. For all studies and species, we did not detect any synergistic interactions between salinity and temperature. Instead, we found individual stressor effects, additive relationships and antagonistic interactions between salinity and temperature. The lack of synergistic interactions in our study suggests that conditions remained below the species’ threshold for either both or one stressor. The overwhelming negative effect of temperature in our study suggests that temperatures surpassed the optimal–thermal threshold, having deleterious effects on both the physiological responses of these fish and survival. These results demonstrate the importance of considering the range of conditions experienced by individuals and whether stressor thresholds have been surpassed or not.

Our results contrast the few previous studies evaluating temperature–salinity interactions on freshwater vertebrates, which generally found synergistic declines in survival and delayed embryonic development and hatch time (Kinne and Kinne, 1962; Tay and Garside, 1974; Rogell *et al.*, 2009; Hopkins *et al.*, 2017). Combined with our results, these studies provide three important findings regarding temperature–salinity interactions. First, while the individual effects of temperature and salinity have been well studied, comparatively few studies have evaluated temperature–salinity interactions on freshwater vertebrates (Hopkins *et al.*, 2017). Second, it is more likely that synergistic interactions stem from both temperature and salinity treatments surpassing species’ stressor thresholds. Lastly, if long-term responses such as survival are altered along the evaluated temperature–salinity gradients, short-term physiological responses of the HPI axis have already been affected. And if the goal of conservation is to protect freshwater species from declining as salinization and water temperatures rise (Iglesias, 2020), the temperature–salinity thresholds and stressor interactions on early warning physiological responses need to be further identified (Tort, 2011).

### Comparing acute and chronic Trout experiments

Evaluating the physiological responses of individuals during acute and chronic exposure to different multiple-stressor combinations can provide insight into their short- and long-term effects (Boonstra, 2013). Our acute (3-day) and chronic (32-day) multiple-stressor experiments for Trout yielded different outcomes. In our acute Trout experiment, baseline cortisol was negatively related to temperature and absolute change in cortisol was positively related to temperature. In addition, stress-induced glucose was best explained by an antagonistic interaction between salinity and temperature, as we predicted. However, under chronic conditions, Trout responses were unrelated to temperature and salinity, suggesting that the surviving individuals had acclimated to the temperature–salinity gradient. But, we must also note that the highest temperatures were reduced from 22°C to 18°C at the beginning of the chronic Trout experiment because all initial Trout died during the acclimation period, when temperatures were 22°C, which is not surprising as 18°C has been documented as *O. clarki*’s critical thermal limit (Mandeville *et al.*, 2019). This suggests that Trout chronically exposed to temperatures above 18°C were unable to acclimate to higher temperature conditions, resulting in negative, inhibitory effects.

### Linking physiology to ONGD

Anthropogenic activities, such as ONGD, which alter habitat and water quality, will variably affect stress physiology for fish and other freshwater organisms. Infrastructure development, decreased vegetative cover and increased chemical contamination are all potential contributors to increased surface-water temperatures and contamination that have been shown to influence markers of stress (Williams *et al.*, 2008; Olmstead *et al.*, 2013). When conditions posed by ONGD acutely emerge, such as pulsed spills (Olmstead *et al.*, 2013; Brittingham *et al.*, 2014), water temperature and other chemical constituents may be more concerning than salinity for freshwater fishes. At higher temperatures, an individual’s ability to mount an appropriate stress response to new stressors may be reduced. In these circumstances, the magnitude of glucose mobilization can depend on temperature–salinity interactions, presenting different outcomes for downstream physiology when handling environmental challenges (e.g. Mazik *et al.*, 1991; Gregory and Wood, 1999; Jentoft *et al.*, 2005; Luz *et al.*, 2008; Chambers, 2011). Contrary to the expectation that altered physiological function would influence life-history investment during such occurrences, overall fish condition seemed to be unaffected. When periods of exposure are chronic, temperature–salinity interactions on HPI activity can be more complex. Cortisol concentrations in particular do not appear to be uniformly regulated, as the directionality of release was co-dependent on temperature and salinity. Basal cortisol is likely to decrease with increased salinity, but concurrent temperature increases can either facilitate or inhibit this pattern of release. Similarly, stress reactivity can be either insensitive to temperature–salinity differences or decline as temperature increases. Species-specific cortisol responses under such conditions do not seem to affect energy strategy for fish growth rate and condition, but if altered physiological state is indeed associated with mortality (Pickering, 1989; Pickering and Pottinger, 1989), surface-water temperature changes and degradation of important refuge habitats should thus be of concern for freshwater organisms within proximity of ONGD.

### Alternative mechanisms

The extent to which stress was elicited by the conditions considered in this study may be reflected by multiple aspects of HPI-axis activity (MacDougall-Shackleton *et al.*, 2019). Of consideration here are not only components of the cortisol response (e.g. receptor populations and affinities, bound and unbound levels; Faught and Vijayan, 2016), but also other primary mediators of allostasis (e.g. catecholamines, cytokines) that can induce blood glucose changes during physiological responses to stress (Perry and Reid, 1993; Reid *et al.*, 1998; Perry and Bernier, 1999; Tort, 2011; Nardocci *et al.*, 2014). Unfortunately, quantification of these mediators is often not feasible due to their rapid mechanistic action during the stress response and analytical capabilities in non-model systems. Although patterns of circulating glucocorticoids sometimes declined with habitat disturbance, even low concentrations can still be relevant for energy mobilization, especially during synergistic action with other allostatic mediators. This scenario may be possible if glucocorticoid release was too expensive, resulting in downregulation of production and/or an adaptive tradeoff with other mediators under chronic conditions. Regardless of the upstream mechanisms by which stressors in these studies were handled, glucose changes indicate a functional stress response occurred when temperature and salinity conditions were altered.

### Other potential stressors and limitations

Temperature and salinity are not the only stressors to consider when evaluating the physiological responses of aquatic animals. In aquatic ecosystems, many other stressors, such as chemical contaminants, dissolved oxygen, pH and food stress could affect fish physiologically. We cannot rule out effects of other contaminants in our field study (Walters *et al.*, 2019), but it is unlikely that additional contaminants affected our laboratory experiments, as all water originated from the same artesian well. Dissolved oxygen was unlikely a stressor in this study, as all dissolved oxygen measurements were considerably greater than any value deemed stressful to aquatic organisms (always >80% and >7.0 mg L^−1^; Davis, 1975). Additionally, altered pH is a concern for aquatic ecosystems, as the capacity for systems to buffer against changes in pH depends on the background concentration of base anions (e.g. HCO_3_^−^; Kaushal *et al.*, 2018). With increased HCO_3_^−^ ions, the system’s buffering capacity and pH should correspondingly increase. However, stress associated with pH changes was likely minimal in our study, as pH remained between 7.9 and 8.7, which is within the range experienced by most freshwater organisms (Fromm, 1980). Food stress was unlikely a stressor in the field (Herring *et al.*, 2011), as benthic macroinvertebrate prey biomass averaged 2966 mg m^−2^ across 40 sites in the study area in 2016 (Walker and Walters, 2019a). While food availability was not an issue, food intake could have influenced our laboratory results as many fish appeared to reduce food consumption in our experiments (although this was not explicitly measured). This could be due to greater stress under laboratory conditions, which can have appetite-suppressing effects on individuals (Bernier and Peter, 2001; Luz *et al.*, 2008, Chambers, 2011).

As survival was low for Sculpin and Sucker at higher temperatures in our laboratory experiments, we must acknowledge potential limitations of the available data and results. While adequate data were available across the salinity ranges, the observed physiological relationships associated with temperature were only evaluated for a small temperature range of ~ 2°C (15–17°C), and therefore, temperature relationships for Sculpin and Sucker in our laboratory experiments should be viewed with caution. Although this constraint potentially limits data interpretation, others have shown that even small differences in temperature can strongly influence the physiological responses of fishes (Meka and McCormick, 2005; Payne *et al.*, 2016).

## Conclusions

Freshwater salinization and rising temperatures are two abiotic stressors expected to intensify across the globe, generating complex interactions with significant implications for freshwater conservation (Pörtner and Farrell, 2008; Hopkins *et al.*, 2017; Iglesias, 2020). Taken together, our field study and laboratory experiments provide a closer look into the interactive effects of temperature and salinity on the physiological responses of freshwater fishes and add to a growing body of research documenting complex multiple-stressor interactions (Jackson *et al.*, 2016; Craig *et al.*, 2017). We showed that temperature and salinity were important physiological mediators for freshwater fish and that the effect of salinity was often temperature dependent, supporting previous notions of temperature-dependent chemical toxicity (Wang *et al.*, 2019). Chronic exposure to elevated salinities, within the ranges evaluated here, appear to have facilitative effects on circulating energy-mobilizing hormones and osmoregulation for freshwater fishes, but the beneficial effects of salinity are weakened as temperatures increased. While it is evident that increased salinization can negatively affect freshwater organisms, we still lack a comprehensive understanding of the osmotic thresholds, physiological and biochemical coping mechanisms, and salinity interactions that allow most species to tolerate salinity stress (Hopkins *et al.*, 2017; Kültz, 2017; Kefford, 2019; Iglesias, 2020). Thus, understanding the interactive effects of multiple stressors and the coping mechanisms for different species are important steps in predicting how individuals, populations and communities will respond to future environmental change.

Resource managers could ameliorate the effects of rising temperatures by increasing or restoring refuge habitats (e.g. deeper pools, more in-stream cover, increased riparian cover) that have been negatively altered, as these habitats can provide important thermal refugia for many species (Torgersen *et al.*, 1999; Caissie, 2006). Furthermore, best management practices (i.e. riparian buffers, silt fences, erosion blankets, revegetation) in conjunction with future land-use change could help maintain contaminant concentrations below negative thresholds for aquatic life. With projected increases in global temperatures (Isaak *et al.*, 2015), these mitigation efforts could help alleviate the effects of temperature-dependent chemical toxicity.

## Funding

This research was funded by the Wyoming Landscape Conservation Initiative and the University of Wyoming’s Biodiversity Institute.

## Conflicts of interest

None declared

## Authors’ Contributions

R.H.W. and A.W.W. developed the research idea. A.W.W. and R.H.W. secured funding. S.S.F. provided laboratory space and refined laboratory procedures. R.H.W., G.D.S. and S.B.H. collected and managed the data. R.H.W. analysed the data and wrote the first manuscript draft. All authors contributed to revisions.

## Supporting information

Upon acceptance, additional Supporting Information may be found online in the supporting information tab for this article.

## Data accessibility

Data associated with this project can be found in the ScienceBase repository upon acceptance (Walker and Walters, 2021).

## Supplementary Material

Supp_CONPHYS_2019_151_coaa107
