# Supplementary material for: Warmer temperatures interact with salinity to weaken physiological facilitation to stress in freshwater fishes
Source: Conserv Physiol. 2020 Dec 15;8(1):coaa107. doi: 10.1093/conphys/coaa107 (PMC7745714; doi:10.1093/conphys/coaa107)
Supplement: Supp_CONPHYS_2019_151_coaa107 [file supp_conphys_2019_151_coaa107.docx]

**Supplementary Information**

Tables

Table S1. List of physiological responses of fishes measured in the field surveys and laboratory experiments. Baseline samples were collected within 3 minutes of capture and stress-induced samples were collected 30 minutes after baseline samples. Absolute change in glucose and cortisol were calculated as the numerical change in baseline and stress-induced samples to represent stress-reactivity.

| Response metric | 2015 | 2016 | 2017 | Experiments |
| --- | --- | --- | --- | --- |
| Baseline |  |  |  |  |
| Cortisol (ng mL^-1^) | X | X | X | X |
| Glucose (mg dL^-1^) | X | X | X | X |
|  |  |  |  |  |
| Stress-induced |  |  |  |  |
| Cortisol (ng mL^-1^) | NA | X | X | X |
| Glucose (mg dL^-1^) | NA | X | X | X |
|  |  |  |  |  |
| Absolute change |  |  |  |  |
| Cortisol (ng mL^-1^) | NA | X | X | X |
| Glucose (mg dL^-1^) | NA | X | X | X |
|  |  |  |  |  |
| Survival | NA | NA | NA | X |
| Instantaneous growth rate | NA | NA | NA | X |
| Condition | X | X | X | X |

*Notes*: X represents responses that were measured, NA represents responses that were not measured.

Table S2. Model results from the field study evaluating the individual and interactive effects of salinity and temperature on the physiological responses of Mottled Sculpin (MSC) and Mountain Sucker (MTS). Data were combined for all years (i.e., 2015, 2016, 2017). Stress-induced samples were not taken in 2015. Interactions between salinity and temperature were tested for each response metric, but interaction results are only reported for models where significant interactions occurred.

| Species | Response metric | Parameter | Beta | SE | t-value | p-value | CL | R^2^m | R^2^c |
| --- | --- | --- | --- | --- | --- | --- | --- | --- | --- |
| MSC | **Baseline cortisol** | **Salinity** | **-0.38** | **0.002** | **-167.60** | **<0.0001** | **-0.38, -0.38** | 0.10 | 0.32 |
|  |  | **Temperature** | **-0.06** | **0.002** | **-28.47** | **<0.0001** | **-0.06, -0.06** |  |  |
|  | Baseline glucose | Salinity | 0.004 | 0.04 | 0.12 | 0.91 | -0.07, 0.07 | 0.001 | 0.09 |
|  |  | Temperature | 0.002 | 0.01 | 0.21 | 0.83 | -0.02, 0.02 |  |  |
|  | Stress-induced cortisol | Salinity | 0.06 | 0.11 | 0.61 | 0.54 | -0.15, 0.27 | 0.01 | 0.12 |
|  |  | Temperature | 0.004 | 0.03 | 0.13 | 0.89 | -0.06, 0.06 |  |  |
|  | **Stress-induced glucose** | **Salinity** | **0.22** | **0.12** | **1.93** | **0.05** | **0.00, 0.45** | 0.28 | 0.38 |
|  |  | **Temperature** | **0.07** | **0.03** | **1.99** | **0.05** | **0.00, 0.14** |  |  |
|  | **Absolute change cortisol** | **Salinity** | **0.01** | **0.004** | **2.72** | **0.007** | **0.003, 0.02** | 0.01 | 0.15 |
|  |  | **Temperature** | **0.32** | **0.12** | **2.63** | **0.009** | **0.08, 0.56** |  |  |
|  |  | **Interaction** | **-0.001** | **0.0003** | **-2.39** | **0.02** | **-0.002, -0.001** |  |  |
|  | **Absolute change glucose** | **Salinity** | **0.31** | **0.14** | **2.16** | **0.03** | **0.03, 0.59** | 0.22 | 0.42 |
|  |  | Temperature | 0.03 | 0.04 | 0.79 | 0.43 | -0.05, 0.11 |  |  |
|  | Condition | Salinity | 0.03 | 0.02 | 1.59 | 0.11 | -0.04, 0.10 | 0.07 | 0.12 |
|  |  | Temperature | 0.004 | 0.01 | 0.74 | 0.46 | -0.12, 0.12 |  |  |
|  |  |  |  |  |  |  |  |  |  |
| MTS | **Baseline cortisol** | **Salinity** | **-0.26** | **0.13** | **-2.06** | **0.04** | **-0.51, -0.01** | 0.06 | 0.24 |
|  |  | **Temperature** | **0.13** | **0.07** | **1.96** | **0.05** | **0.00, 0.26** |  |  |
|  | Baseline glucose | Salinity | -0.09 | 0.06 | -1.41 | 0.16 | -0.22, 0.04 | 0.09 | 0.29 |
|  |  | Temperature | 0.001 | 0.04 | 0.02 | 0.99 | -0.07, 0.07 |  |  |
|  | Stress-induced cortisol | Salinity | 0.06 | 0.08 | 0.70 | 0.48 | -0.09, 0.21 | 0.03 | 0.12 |
|  |  | Temperature | 0.03 | 0.04 | 0.71 | 0.48 | -0.06, 0.12 |  |  |
|  | Stress-induced glucose | Salinity | 0.04 | 0.08 | 0.50 | 0.62 | -0.12, 0.20 | 0.20 | 0.42 |
|  |  | Temperature | 0.07 | 0.05 | 1.41 | 0.16 | -0.02, 0.16 |  |  |
|  | **Absolute change cortisol** | **Salinity** | **0.37** | **0.005** | **78.27** | **<0.0001** | **0.36, 0.38** | 0.03 | 0.17 |
|  |  | **Temperature** | **0.01** | **0.004** | **2.03** | **0.04** | **0.00, 0.02** |  |  |
|  |  | **Interaction** | **-0.03** | **0.004** | **-5.92** | **<0.0001** | **-0.04, -0.02** |  |  |
|  | Absolute change glucose | Salinity | 0.02 | 0.09 | 0.19 | 0.85 | -0.16, 0.20 | 0.01 | 0.16 |
|  |  | Temperature | 0.02 | 0.05 | 0.43 | 0.67 | -0.08, 0.12 |  |  |
|  | Condition | Salinity | 0.01 | 0.02 | 0.67 | 0.50 | -0.02, 0.04 | 0.01 | 0.08 |
|  |  | Temperature | 0.002 | 0.01 | 0.24 | 0.81 | -0.02, 0.02 |  |  |

*Notes*: Salinity was measured as specific conductivity (μS_25˚C_ /cm) and temperature was measured in °C. Bolded response metrics reflect significant relationships. Baseline samples were collected within 3 minutes of capture and stress-induced samples were collected 30 minutes after baseline samples. CL = confidence limits. R^2^m and R^2^c are marginal and conditional R2, respectively. All models included a random intercept term for site nested within year.

Table S3. Model results from acute (3-day) and chronic (32-day) experiments evaluating the individual and interactive effects of salinity and temperature on physiological responses of Colorado River Cutthroat Trout. Interactions between salinity and temperature were tested for each response variable, but interaction results are only reported for models where significant interactions occurred.

| Experiment | Response variable | Parameter | Beta | SE | t-value | p-value | CL | R^2^m | R^2^c |
| --- | --- | --- | --- | --- | --- | --- | --- | --- | --- |
| Acute | **Baseline cortisol** | Salinity | 0.03 | 0.09 | 0.34 | 0.74 | -0.14, 0.20 | 0.21 | 0.32 |
|  |  | **Temperature** | **-0.08** | **0.03** | **-2.19** | **0.03** | **-0.15, -0.01** |  |  |
|  | Baseline glucose | Salinity | 0.04 | 0.05 | 0.83 | 0.41 | -0.46, 0.54 | 0.07 | 0.29 |
|  |  | Temperature | -0.01 | 0.02 | -0.75 | 0.46 | -0.03, 0.01 |  |  |
|  | Stress-induced cortisol | Salinity | 0.004 | 0.03 | 0.11 | 0.91 | -0.07, 0.07 | 0.09 | 0.11 |
|  |  | Temperature | -0.02 | 0.01 | -1.90 | 0.06 | -0.04, 0.00 |  |  |
|  | **Stress-induced glucose** | **Salinity** | **0.73** | **0.19** | **3.93** | **<0.0001** | **0.37, 1.09** | 0.34 | 0.34 |
|  |  | **Temperature** | **-0.03** | **0.01** | **-2.89** | **0.004** | **-0.05, -0.01** |  |  |
|  |  | **Interaction** | **-0.04** | **0.01** | **-3.90** | **<0.0001** | **-0.06, -0.02** |  |  |
|  | **Absolute change cortisol** | Salinity | -0.14 | 0.01 | -1.12 | 0.26 | -0.39, 0.11 | 0.15 | 0.15 |
|  |  | **Temperature** | **0.13** | **0.05** | **2.67** | **0.01** | **0.04, 0.22** |  |  |
|  | Absolute change glucose | Salinity | -0.02 | 0.06 | -0.40 | 0.69 | -0.14, 0.10 | 0.06 | 0.06 |
|  |  | Temperature | -0.03 | 0.02 | -1.12 | 0.26 | -0.08, 0.02 |  |  |
|  | Condition | Salinity | -0.004 | 0.02 | -0.24 | 0.81 | -0.03, 0.03 | 0.03 | 0.04 |
|  |  | Temperature | -0.01 | 0.01 | -1.34 | 0.18 | -0.02, 0.00 |  |  |
|  |  |  |  |  |  |  |  |  |  |
| Chronic | Baseline cortisol | Salinity | 0.06 | 0.20 | 0.32 | 0.75 | -0.33, 0.45 | 0.01 | 0.23 |
|  |  | Temperature | -0.04 | 0.09 | -0.41 | 0.69 | -0.22, 0.14 |  |  |
|  | Baseline glucose | Salinity | -0.03 | 0.03 | -1.16 | 0.25 | -0.08, 0.02 | 0.11 | 0.32 |
|  |  | Temperature | -0.01 | 0.01 | -0.65 | 0.52 | -0.03, 0.01 |  |  |
|  | Stress-induced cortisol | Salinity | 0.02 | 0.02 | 1.12 | 0.26 | -0.02, 0.06 | 0.07 | 0.13 |
|  |  | Temperature | -0.01 | 0.01 | -0.84 | 0.40 | -0.03, 0.01 |  |  |
|  | Stress-induced glucose | Salinity | -0.05 | 0.04 | -1.21 | 0.23 | -0.13, 0.03 | 0.04 | 0.06 |
|  |  | Temperature | 0.01 | 0.02 | 0.37 | 0.71 | -0.02, 0.03 |  |  |
|  | Absolute change cortisol | Salinity | 0.08 | 0.01 | 0.92 | 0.36 | -0.10 0.26 | 0.05 | 0.11 |
|  |  | Temperature | -0.01 | 0.04 | -0.35 | 0.72 | -0.09, 0.07 |  |  |
|  | Absolute change glucose | Salinity | -0.05 | 0.18 | -0.28 | 0.78 | -0.04, 0.03 | 0.01 | 0.01 |
|  |  | Temperature | 0.02 | 0.07 | 0.34 | 0.73 | -0.12, 0.16 |  |  |
|  | Condition | Salinity | 0.01 | 0.03 | 0.19 | 0.85 | -0.05, 0.07 | 0.16 | 0.34 |
|  |  | Temperature | -0.03 | 0.01 | -1.88 | 0.06 | -0.06, 0.00 |  |  |
|  | Growth rate | Salinity | -0.005 | -0.09 | 0.93 | 0.12 | -0.13, 0.12 | 0.01 | 0.01 |
|  |  | Temperature | -0.006 | 0.02 | -0.22 | 0.83 | -0.07, 0.05 |  |  |
|  | **Survival** | Salinity | 0.004 | 0.009 | 0.42 | 0.68 | -0.02, 0.02 | 0.37 | 0.68 |
|  |  | **Temperature** | **-0.07** | **0.02** | **-2.89** | **0.02** | **-0.12, -0.01** |  |  |

*Notes*: Salinity was measured as specific conductivity (μS_25˚C_ /cm) and temperature was measured in °C. Bolded response metrics reflect significant relationships. Baseline samples were collected within 3 minutes of capture and stress-induced samples were collected 30 minutes after baseline samples. CL = confidence limits. R^2^m and R^2^c are marginal and conditional R2, respectively. All models included a random intercept term for treatment.

Table S4. Model results from chronic (32-day) experiments evaluating the individual and interactive effects of Salinity and temperature on physiological responses of Mottled Sculpin (MSC) and Mountain Sucker (MTS). Interactions between salinity and temperature were tested for each response variable, but interaction results are only reported for models where significant interactions occurred.

| Species | Response variable | Parameter | Beta | SE | t-value | p-value | CL | R^2^m | R^2^c |
| --- | --- | --- | --- | --- | --- | --- | --- | --- | --- |
| MSC | **Survival** | Salinity | -0.01 | 0.01 | -0.80 | 0.44 | -0.04, 0.02 | 0.37 | 0.48 |
|  |  | **Temperature** | **-0.09** | **0.03** | **-2.78** | **0.02** | **-0.16, -0.02** |  |  |
|  | Growth rate | Salinity | 0.04 | 0.08 | 0.51 | 0.61 | -0.13, 0.21 | 0.05 | 0.05 |
|  |  | Temperature | 0.04 | 0.06 | 0.69 | 0.49 | -0.08, 0.16 |  |  |
|  | Condition | Salinity | -0.0002 | 0.03 | -0.01 | 0.99 | -0.06, 0.06 | 0.04 | 0.05 |
|  |  | Temperature | -0.02 | 0.02 | -0.77 | 0.44 | -0.42, 0.38 |  |  |
|  | Baseline cortisol | Salinity | -0.45 | 0.30 | -1.48 | 0.14 | -1.05, 0.15 | 0.37 | 0.37 |
|  |  | Temperature | 0.14 | 0.17 | 0.88 | 0.38 | -0.19, 0.47 |  |  |
|  | Baseline glucose | Salinity | -0.003 | 0.09 | -0.04 | 0.97 | -0.18, 0.18 | 0.19 | 0.33 |
|  |  | Temperature | 0.08 | 0.05 | 1.61 | 0.11 | -0.02, 0.18 |  |  |
|  | Stress-induced cortisol | Salinity | 0.01 | 0.09 | 0.17 | 0.87 | -0.16, 0.18 | 0.03 | 0.03 |
|  |  | Temperature | 0.03 | 0.06 | 0.63 | 0.63 | -0.09, 0.15 |  |  |
|  | Stress-induced glucose | Salinity | -0.04 | 0.20 | -0.19 | 0.85 | -0.43, 0.35 | 0.04 | 0.36 |
|  |  | Temperature | 0.04 | 0.09 | 0.42 | 0.67 | -0.14, 0.22 |  |  |
|  | **Absolute change cortisol** | Salinity | 0.08 | 0.25 | 0.32 | 0.75 | -0.41, 0.57 | 0.76 | 0.76 |
|  |  | **Temperature** | **-0.74** | **0.17** | **-4.29** | **<0.0001** | **-1.08, -0.40** |  |  |
|  | Absolute change glucose | Salinity | -0.07 | 0.16 | -0.43 | 0.67 | -0.38, 0.24 | 0.02 | 0.02 |
|  |  | Temperature | -0.03 | 0.11 | -0.24 | 0.81 | -0.25, 0.19 |  |  |
|  |  |  |  |  |  |  |  |  |  |
| MTS | **Survival** | Salinity | 0.003 | 0.002 | 1.05 | 0.32 | -0.003, 0.008 | 0.97 | 0.98 |
|  |  | **Temperature** | **-0.18** | **0.006** | **-28.49** | **<0.0001** | **-0.18, -0.13** |  |  |
|  | Growth rate | Salinity | -0.01 | 0.04 | -0.12 | 0.90 | -0.1, 0.08 | 0.001 | 0.001 |
|  |  | Temperature | -0.09 | 0.09 | 1.00 | 0.32 | -0.26, 0.08 |  |  |
|  | Condition | Salinity | -0.06 | 0.10 | -0.66 | 0.51 | -0.25, 0.13 | 0.05 | 0.05 |
|  |  | Temperature | -0.15 | 0.19 | -0.80 | 0.42 | -0.51, 0.21 |  |  |
|  | **Baseline cortisol** | **Salinity** | **-27.09** | **13.22** | **-2.05** | **0.04** | **-53.01, -1.17** | 0.38 | 0.38 |
|  |  | Temperature | -0.53 | 0.96 | -0.55 | 0.58 | -2.40, 1.34 |  |  |
|  |  | **Interaction** | **1.72** | **0.85** | **2.02** | **0.04** | **0.05, 3.39** |  |  |
|  | Baseline glucose | Salinity | 0.17 | 0.15 | 1.18 | 0.24 | -0.11, 0.45 | 0.14 | 0.32 |
|  |  | Temperature | 0.04 | 0.23 | 0.15 | 0.88 | -0.42, 0.50 |  |  |
|  | **Stress-induced cortisol** | Salinity | -0.24 | 0.23 | -1.05 | 0.29 | -0.68, 0.20 | 0.50 | 0.56 |
|  |  | **Temperature** | **-1.05** | **0.45** | **-2.34** | **0.02** | **-1.93, -0.17** |  |  |
|  | Stress-induced glucose* | Salinity | -0.01 | 0.16 | -0.06 | 0.95 | -0.31, 0.29 | 0.001 | 0.23 |
|  |  | Temperature | -0.09 | 0.30 | -0.32 | 0.75 | -0.67, 0.49 | 0.01 | 0.27 |
|  | **Absolute change cortisol** | Salinity | -0.28 | 0.22 | -1.32 | 0.19 | -0.70, 0.14 | 0.48 | 0.86 |
|  |  | **Temperature** | **-1.27** | **0.44** | **-2.91** | **0.004** | **-2.13, -0.41** |  |  |
|  | Absolute change glucose | Salinity | -0.13 | 0.28 | -0.44 | 0.66 | -0.68, 0.42 | 0.04 | 0.19 |
|  |  | Temperature | -0.02 | 0.54 | -0.04 | 0.97 | -1.08, 1.04 |  |  |

*Notes*: * reflect models that would not converge with both parameters, thus values reflect a model with individual parameters. Bolded response metrics reflect significant relationships. Salinity was measured as specific conductivity (μS_25˚C_ /cm) and temperature was measured in °C. Baseline samples were collected within 3 minutes of capture and stress-induced samples were collected 30 minutes after baseline samples. CL = confidence limits. R^2^m and R^2^c are marginal and conditional R^2^, respectively. All models included a random intercept term for treatment.

Figures

Figure S1. Map of study area in the Wyoming Range in the Upper Green River Basin, Wyoming depicting sample locations where Mottled Sculpin and Mountain Sucker were collected to evaluate the physiological responses associated with oil and natural gas development between 2015 and 2017. The white box represents the state of Wyoming. Latitude and longitudes for all sites can be found in Table 2. Percent catchment-level land-use change associated with ONGD ranged from 0.0% to 9.2%.


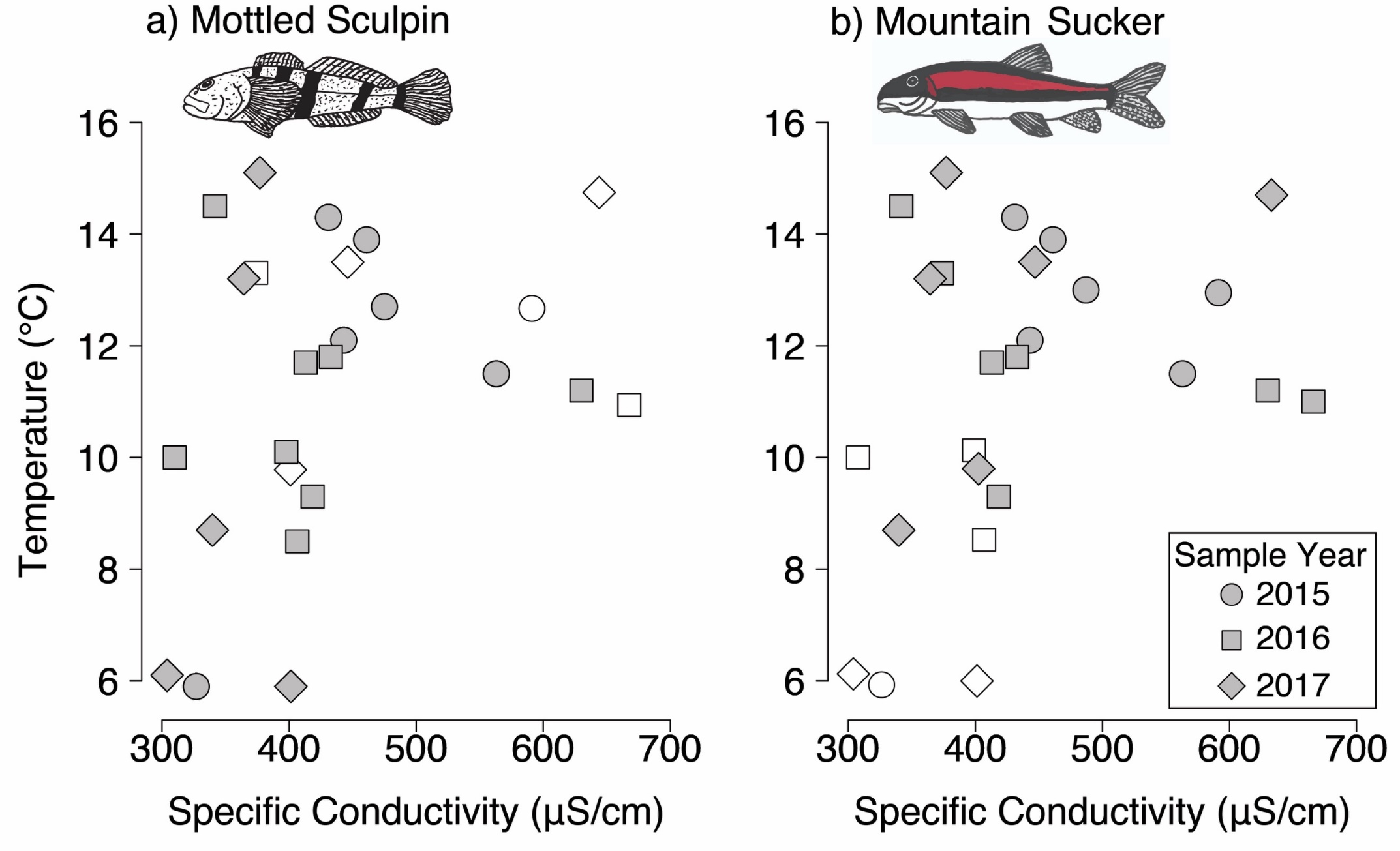


Figure S2. Distribution of *in-situ* salinity and temperature measurements from streams in the Wyoming Range, Wyoming in August of 2015, 2016, and 2017 where Mottled Sculpin (a) and Mountain Sucker (b) were collected. Grey points represent years and sample locations where fish were sampled, while white points represent years and sample locations where fish were not sampled. White points reflect sample locations where fish numbers were too low and/or fish were too small to collect blood from.


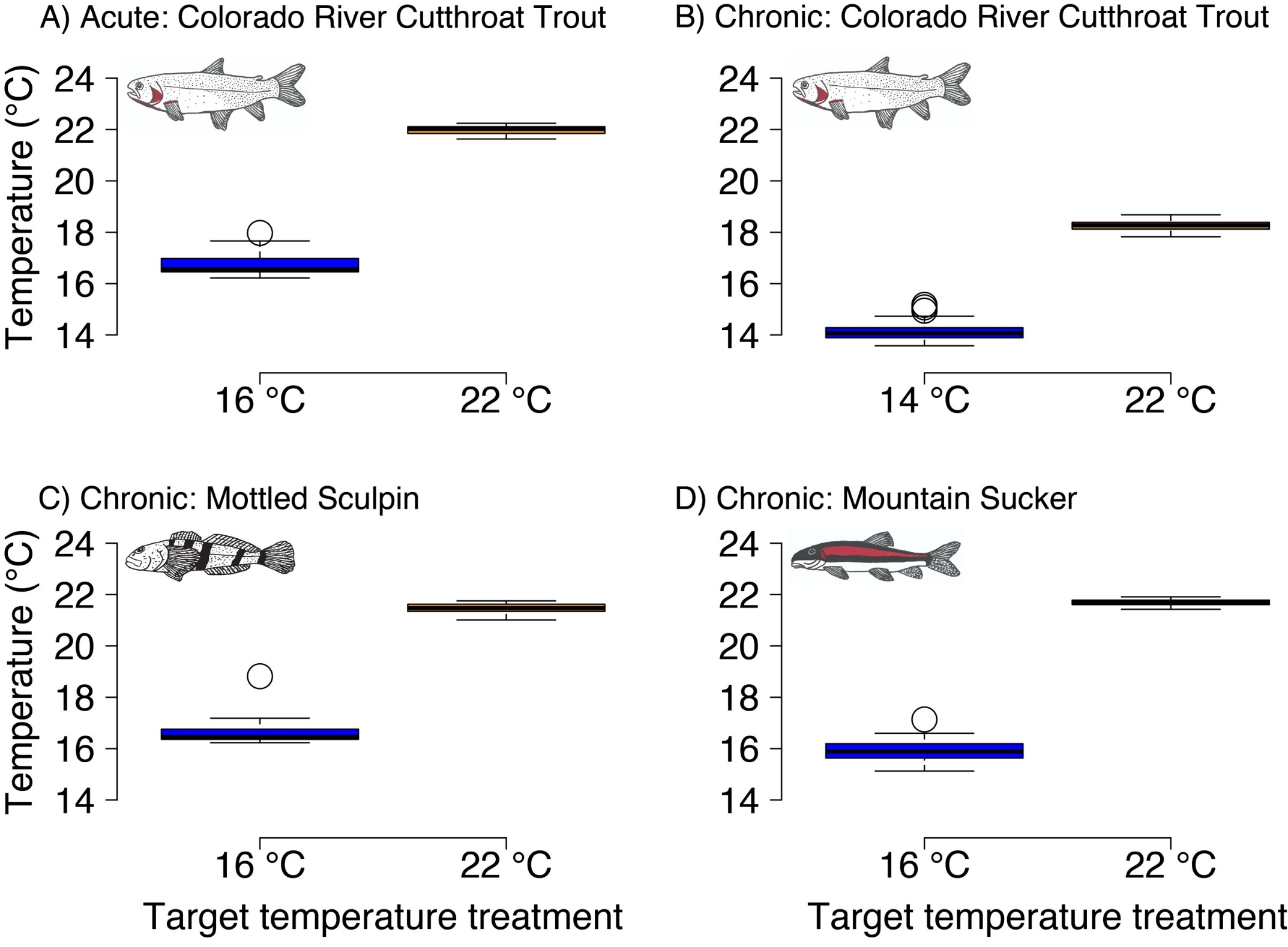


Figure S3. Boxplot showing range of temperatures measured for each target temperature treatment for Colorado River Cutthroat Trout (A and B), Mottled Sculpin (C), and Mountain Sucker (D) during the acute (3-day) and chronic (32-day) multiple-stressor experiments. Measured temperatures were significantly different for all experiments. Each target temperature treatment reflects the ranges of 24 replicate aquaria.


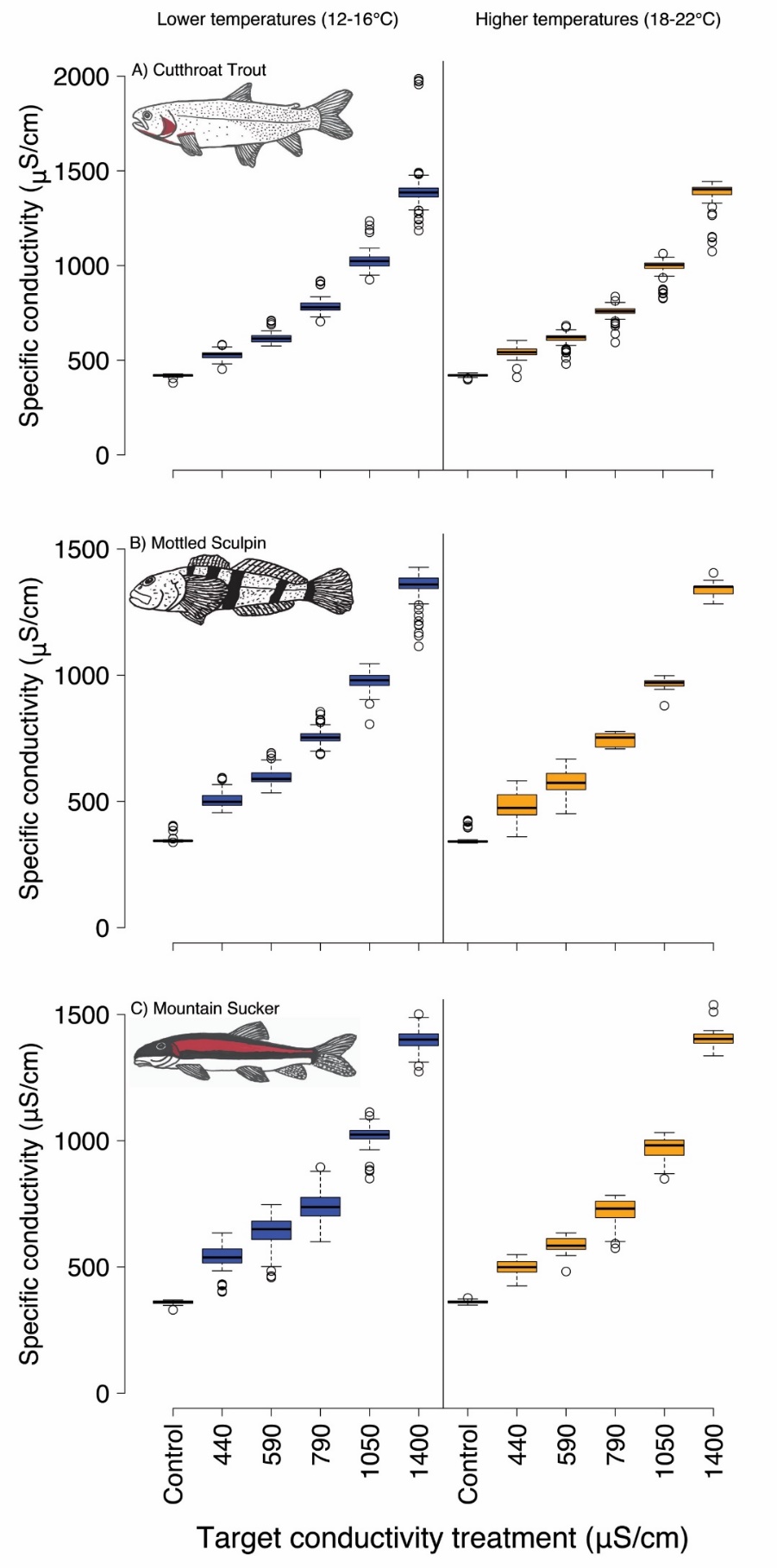


Figure S4. Boxplot showing range of salinity measured for each target salinity treatment for Colorado River Cutthroat Trout (A; CRCT), Mottled Sculpin (B), and Mountain Sucker (C) during the chronic (32-day) multiple-stressor experiments. The left column represents salinity at lower temperature treatments and the right column represents salinity at higher temperature treatments. Each target salinity treatment reflects the ranges of four replicate aquaria. Data for each species represents a gradient of 12 different salinity-temperature treatment. Measured salinities for the acute (32-day) CRCT experiment were within the same range as the chronic CRCT experiment (not shown).


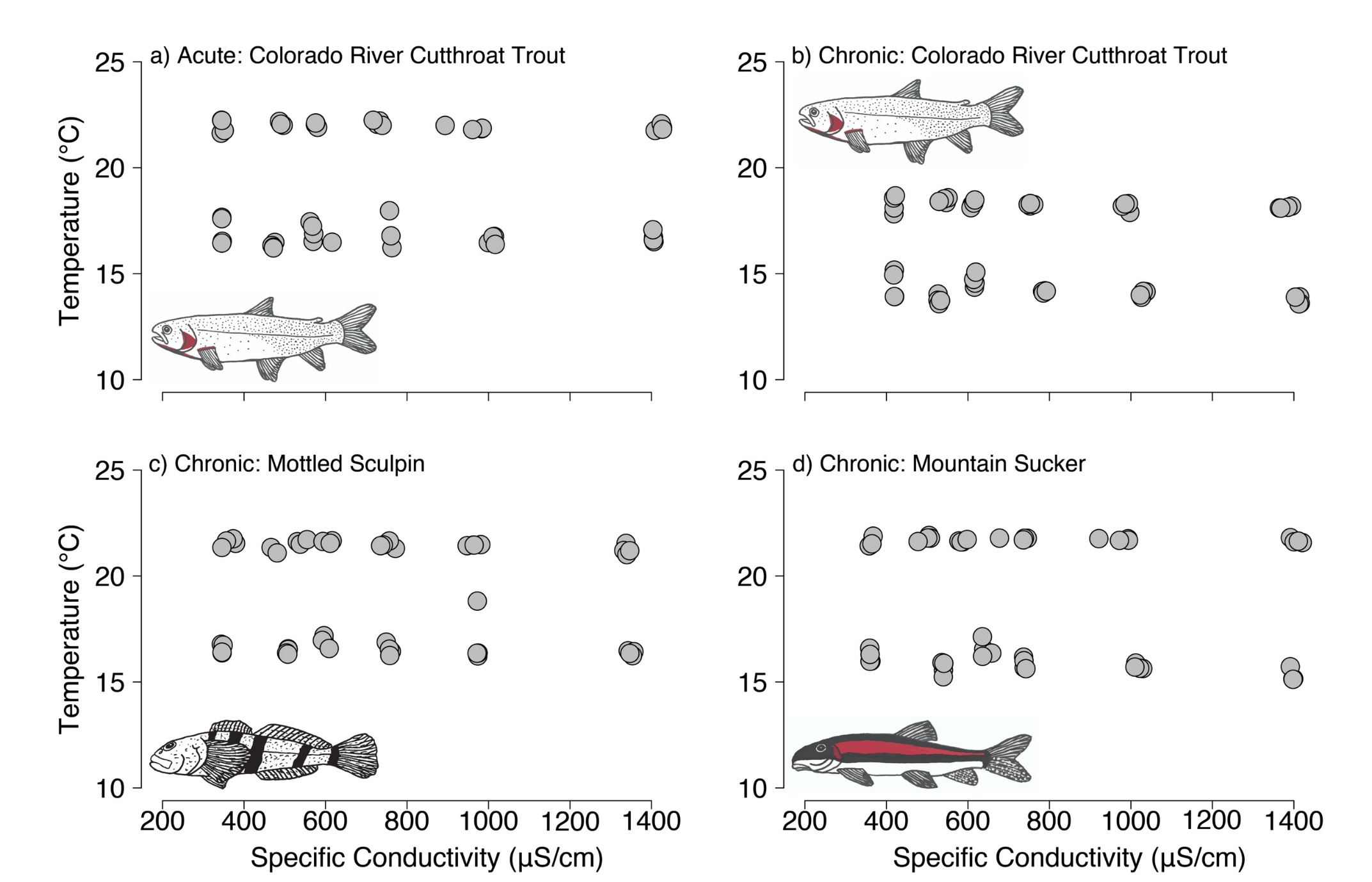


Figure S5. Distribution of mean salinity (conductivity (μS_25˚C_ /cm)) and temperature (°C) gradients experienced by Colorado River Cutthroat Trout (a and b), Mottled Sculpin (c), and Mountain Sucker (d) during acute (3-day) and chronic (32-day) multiple-stressor experiments. Each point represents an individual aquarium, representing four replicates per salinity-temperature treatment (48 aquaria total). Values reflect added NaHCO_3_ with the lowest salinity in each experiment represents the control treatment, where no NaHCO_3_ was added. All other specific salinity values reflect added NaHCO_3_^-^ with a 75% dilution factor.
